# On Ontological Alternatives to Bohmian Mechanics

**DOI:** 10.3390/e20060474

**Published:** 2018-06-19

**Authors:** Thomas Filk

**Affiliations:** 1 Institute for Physics, University of Freiburg, Hermann-Herder-Str. 3, D-79104 Freiburg, Germany; thomas.filk@physik.uni-freiburg.de; 2 Parmenides Foundation for the Study of Thinking, D-82049 Munich-Pullach, Germany

**Keywords:** relational space, relational interpretation of quantum mechanics, measurement problem, non-locality

## Abstract

The article describes an interpretation of the mathematical formalism of standard quantum mechanics in terms of relations. In particular, the wave function ψ(x) is interpreted as a complex-valued relation between an entity (often called “particle”) and a second entity *x* (often called “spatial point”). Such complex-valued relations can also be formulated for classical physical systems. Entanglement is interpreted as a relation between two entities (particles or properties of particles). Such relations define the concept of “being next to each other”, which implies that entangled entities are close to each other, even though they might appear to be far away with respect to a classical background space. However, when space is also considered to be a network of relations (of which the classical background space is a large-scale continuum limit), such nearest neighbor configurations are possible. The measurement problem is discussed from the perspective of this interpretation. It should be emphasized that this interpretation is not meant to be a serious attempt to describe *the* ontology of our world, but its purpose is to make it obvious that, besides Bohmian mechanics, presumably many other ontological interpretations of quantum theory exist.

## 1. Introduction

Bohmian mechanics [1] became a refuge for scientists and philosophers of science in search of an interpretation of quantum theory, which offers a consistent ontology (for an introduction to Bohmian mechanics, see e.g., [2,3,4]). Its experimentally verifiable predictions agree with standard quantum mechanics almost by construction, but its ontology is based on degrees of freedom which, again by construction, are not directly accessible to experiments. There are other models that can offer an ontology, like the so-called collapse models of Ghirardi, Rimini and Weber [5,6] (for relativistic extensions see, e.g., [7]); however, these models predict a deviation from quantum mechanics for mesoscopic systems, for which the influence of collapse centers cannot be neglected. Similar models by Karolyhazy [8] and Penrose [9,10] attribute the physical collapse of the wave function to an influence of gravity, which effectively leads to similar deviations from quantum theory as the collapse models. Sooner or later, we should be able to decide by experiment whether or not these collapse models are correct. However, any experimental disagreement with Bohmian mechanics would also be a disagreement with standard quantum theory (at least for those observables for which measurable expectation values can be calculated in quantum theory).

David Bohm himself did not consider his model of quantum theory as *the* ontology of the world, but he emphasized on several occasions that this model is *about ontology* [11]. (Similar remarks can also be found in the first chapter of [12].) He proved that an ontological formulation of quantum theory is possible, despite so-called “no-go”-theorems by von Neumann [13] and others. The price which Bohmian mechanics has to pay is a non-local ‘influenciability’, not associated with energy or physical information (in the sense that this influenciabilty can be used to transmit signals), and the introduction of unobservable degrees of freedom. Whether or not non-local influenciability exists also for other interpretations of quantum theory is a matter of debate, however, as Bohmian mechanics defines an ontology, this influenciability has an ontological basis, in contrast, e.g., to interpretations of quantum theory, where quantum states represent our knowledge about the world (an example is QBism; for a review, see [14]; for criticism, see [15]).

Bohmian mechanics is based on an ontology that is close to classical Newtonian mechanics in the sense that there exist particles that propagate along well-defined trajectories, and, in addition, there exist fields (the so-called guidance fields or pilot waves), which can be derived from the solutions of Schrödinger’s equation and which play a similar role as potentials in classical physics.

In this article, I will outline an interpretation of the quantum formalism which differs in essential ways from Bohmian mechanics: there are no particles that follow trajectories, but there are entities that have relations to spatial points, and a change of these relations leads to the impression of motion. Like Bohmian mechanics, this ontology is based on an unchanged quantum formalism, and, therefore, leads to the same predictions as quantum theory proper. However, I should emphasize that this difference in interpretations refers to Bohmian mechanics in its standard form (like, e.g., in [2,3]), not necessarily to the implicate order, which, according to the later ideas of Bohm, may underlie both quantum theory and relativity.

At first sight, some parts of this ontological model may look very artificial and seem far from being “natural”. However, the message is not to sell this as the “right” ontology. The message is that, apart from Bohmian mechanics, other ontological interpretations of quantum theory are possible (I am convinced that there are many more models that “do the job”) and that, therefore, none of these interpretations can claim to be *the* ontological interpretation of quantum theory.

Many details of this ontology have not been worked out completely, but it should be obvious that this is possible in principle (often in many different ways). Again, this is because my aim is not to propose a complete model but because I want to convince the reader that such models are possible. Some “mechanisms” have been copied from neural network theory, which is one of my research fields, but I am convinced that almost any field (engineering, biology, psychology, sociology, maybe even chemical networks, fluid dynamics, complex systems theory, electronics, etc.) can give rise to the mechanisms needed to fill the gaps between the general concepts and concrete realizations. For me, the lesson is twofold: (1) we shouldn’t give up until we find a really “natural” and maybe even “beautiful” interpretation, (2) until then we can stick to the cooking recipe of quantum mechanics in the firm knowledge that ontological interpretations do exist.

The next section contains a very brief summary of the ingredients of Bohmian mechanics as compared to the relational model of this article. Most of the rest can be considered an elaboration on these ingredients. In Section 3, I will introduce the notion of a relational space and a relational location. In this section, I will also describe the necessary generalizations that are needed to apply this picture to quantum theory. Section 4 describes this relational framework for one-particle quantum mechanics, while, in Section 5, I will extend this formalism to many-particle quantum systems. Section 6 indicates what a generalization to quantum field theory may look like. A brief summary concludes this article.

Finally, I would like to mention that there exists a “relational interpretation of quantum theory”, mainly due to Carlo Rovelli [16]. There may be parallels, but, according to my understanding of Rovelli’s theory, at least the starting points are different. The notion of “relational” in Rovelli’s interpretation refers more to what is observed *in relation to* an observer. In the interpretation given here, “relational” refers to objective, observer independent relations between certain entities. In order to distinguish my relational interpretation of quantum mechanics from Rovelli’s interpretation, I will sometimes refer to my interpretation as the “micro-relational interpretation”. Some of the ideas presented in this article have been published earlier (see [17,18,19]) but are presented from slightly different perspectives. In particular, this article includes more explicit examples from every-day life or classical physics (like neural networks) that exhibit similar relational structures. This includes examples for complex relations, entanglement and “collapse”.

## 2. Bohmian Mechanics and the Microrelational Interpretation in a Nutshell

This section is a brief summary of the essential features of Bohmian mechanics on the one hand and the microrelational interpretation on the other. The aim is to emphasize the differences in both ontologies. Furthermore, it is minimalistic in the sense that I list the necessary features of both ontologies in order to agree with standard quantum theory.

In Bohmian mechanics, the wave function ψ(x) *and* the particle haven an ontological character. The wave function satisfies Schrödinger’s equation. The particle is guided by the field and its trajectory x(t) is such that the probability density of finding the particle at a particular location *x* is proportional to ${\left|\psi \left(x\right)\right|}^{2}$. Bohmian mechanics specifies an equation of motion for this trajectory in terms of the wave function and it can be shown that, for these dynamics, the probability requirement holds (at least for quite general initial conditions). However, this particular equation of motion, which is deterministic and can be derived from a polar decomposition of the wave function, is not the only possibility to satisfy the probability requirement.

For multi-particle systems, the wave function is defined over configuration space. The trajectory of several particles becomes the trajectory of a single point in configuration space. In this way, entanglement is automatically built into the model.

In the microrelational interpretation, particles exist as entities that can have relations to other entities. In particular, they do not (for a given moment in time) have a fixed location in space, but their “location” is specified by a complex-valued relation, which is defined by the wave function ψ(x). This wave function also satisfies Schrödinger’s equation. Particles don’t move, but their relations to spatial entities change. Probing the relation of a particle with a particular point *x* (or volume in space) induces an “all-or-nothing” change: with a probability (density) proportional to ${\left|\psi \left(x\right)\right|}^{2}$, the relation becomes 1 (the particle “is” at this particular point or in that volume); or it becomes zero for this point. This behavior may sound artificial, but, in Section 4.5, I discuss an every-day example that exhibits similar properties.

The microrelational interpretation doesn’t need space (or space-time) to be relational, but defining also relations between spatial entities and letting the topological and metrical properties of space become large scale features of this relational space makes the whole picture more coherent.

Entanglement is interpreted as a relation between two particles such that the relations of one particle to spatial entities depend on the relations of the other particle to spatial entities. This leads to three different types of relations: relations among spatial entities (leading to, say, Euclidean space), relations between particles and spatial entities (they are defined by the wave function), and relations among particles (which lead to entanglement). Whether these three types of relations are fundamentally different (or just different manifestations of the same type of fundamental relation) is left open. The interpretation of entanglement as a ‘nearest neighbor’ relation makes it possible to keep locality (in a sense defined in Section 5.3), which in my opinion is a charming feature of a relational interpretation.

One tentative idea is that the relations underlying entanglement are the most fundamental ones (these relations may not be the same as the entanglement relations used in standard quantum mechanics, but relations that allow the two entities to share certain information such that in measurements quantum correlations are observed). The two other relations are then effective large scale limits of this fundamental relation.

The whole approach is not to be understood as a fully worked out theory or model but rather as a “program”. The idea is not to fill out the details—I am convinced that this can be done in many different ways. The question is whether there are fundamental or logical limitations that would make such a program impossible. If not, many ontological interpretations of quantum theory may coexist and (unless we are able to probe space or space-time at the fundamental level) are indistinguishable with respect to their experimental predictions.

## 3. Relational Entities

Starting from a mathematical definition of “relation”, I first introduce the notion of a relational space. This concept was favored by many philosophers of science, amongst others by Descartes [20] and Leibniz [21]. It is the antipode to the notion of an absolute space that is viewed like a “stage” for matter and which was favored, amongst others, by Newton. Finally, I discuss the notion of “being somewhere” with respect to a relational space, and I will extend this notion to complex-valued relations.

### 3.1. Mathematical Relations

**Figure 1:**
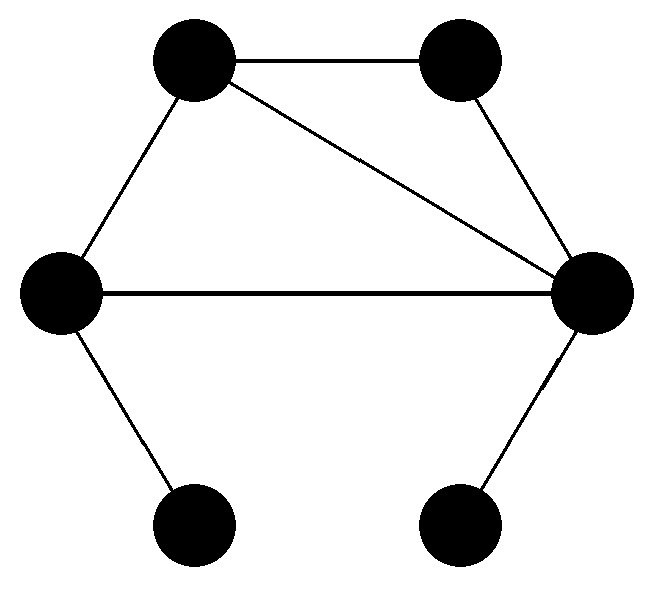
A set with six points and undirected relations. In this case, the relations allow a unique identification of the elements.

Mathematically, a relation *E* on a set *V* is a subset of V×V. We can represent *V* by a set of points and the relations *E* by (directed) lines. Any relation can be represented by a (directed) graph. If the relation *E* is symmetric (i.e., (a,b)∈E⇒(b,a)∈E for all a,b∈V), it can be represented by an undirected graph (Figure 1). Any relation can be expressed by its adjacency matrix:

(1) $$A_{xy}=\left\{\begin{array}{cc} 1,\; & \mathrm{if}\;(y,x)\in E,\\ 0,\; & \mathrm{otherwise}. \end{array}\right.$$

In the following, I will exclude reflexive relations, i.e., the diagonal elements of the adjacency matrix are zero. This leaves us with $2^{V(V-1)/2}$ different undirected relational sets or $2^{V(V-1)}$ directed sets.

In Figure 1, all elements are uniquely specified by these relations. For example, such a specification could be:

- One node has four neighbors.
- One node has two neighbors.
- One node has only one neighbor, which has three neighbors.
- One node has only one neighbor, which has four neighbors.
- One node has three neighbors of which one has one neighbor.
- One node has three neighbors of which one has two neighbors.

Actually, this is the smallest non-trivial (having more than one node) connected (each node can be connected to each other node by a path along existing lines) undirected relational set for which this is possible. The requirement for this being possible is that the graph has no symmetry. This means that there exists no permutation of vertices that leaves the graph unchanged. In other words, there exists no permutation matrix acting on the elements of *V* that commutes with *A*. The probability for a random graph having a symmetry, i.e., that some of its elements are not uniquely identifiable by the relational properties of the graph, gets smaller with an increasing number of vertices.

### 3.2. Relational Space

A relational space is defined as a set *V* of elements, which will be called spatial points, and an undirected relation *E*, which defines a “nearest neighbor” relation for spatial points. “Nearest neighbor” is not to be understood as “next to each other in an already existing space”. Well-known examples of relational spaces are “co-authorship networks” in a scientific community (two scientists being “nearest neighbors” if they are co-authors of a scientific article), semantic networks (two words being related if they are listed as synonyms in a dictionary), protein networks in organisms (e.g., two proteins being related if a chemical reaction in this organism involves both proteins), etc. (for more examples and the statistical properties of such networks, see, e.g., [22]).

While in general such relational spaces are called networks, I will use the term “space” if this network is meant to be a model of the underlying structure of our three-dimensional space. In addition, even though I will refer to the elements of this space as “spatial points”, the notion of a point should not be taken literally because, in a network, the shape of an object is defined by its relations. In addition, the representation of relational spaces by undirected graphs, for which the spatial points are depicted as nodes (often in a plane) with lines connecting these nodes, serves merely as an illustration of the relations. The location of these nodes as points in a plane has no intrinsic meaning whatsoever.

There are several ways to define a distance between the elements of a relational set. One possibility is the “length of the shortest connecting path”, i.e., the minimal number of nearest neighbor steps which are needed to connect the two points. This is often called the mathematical distance. Another possibility is the “propagator distance” [23,24] that is motivated by physical arguments (the propagation of particles in a scaling limit) and involves statistical sums over all paths connecting two points. Once a distance has been defined for any two points, the dimension *d* of a point a∈V is defined by the relation $\mathrm{Vol}\left(a\right)\propto r{\left(a\right)}^{d}$, where Vol(a) is the number of nodes within a distance of r(a) from a point *a*. If this dimension is independent of the point *a*, we call it the dimension of the graph. The concept of a scalar curvature can be defined by deviations from this formula (in [25], geometrical concepts have been investigated in more detail for such relational spaces). Of course, these concepts are only meaningful for very large graphs (ideally for V→∞). We assume that such a relational space—almost flat and of dimension three—is given. The extension of this concept to relational space-time sets—so-called causal sets, for which the elements are events—will be discussed in Section 6.

### 3.3. “To Be” in a Relational Space

Having discussed the notion of a relational space, I now discuss the meaning of “Where *is* an object?” In a relational space, the location of a spatial point is given by the set of relations it has to all other spatial points. However, when we want to specify the location of a non-spatial object (e.g., an entity which we might associate with a charged particle, i.e., an entity which is different from a spatial point), we have more options. In particular, the type of relations that this entity has with spatial entities will, at least in general, be different from the type of relations between spatial entities.

**Figure 2:**
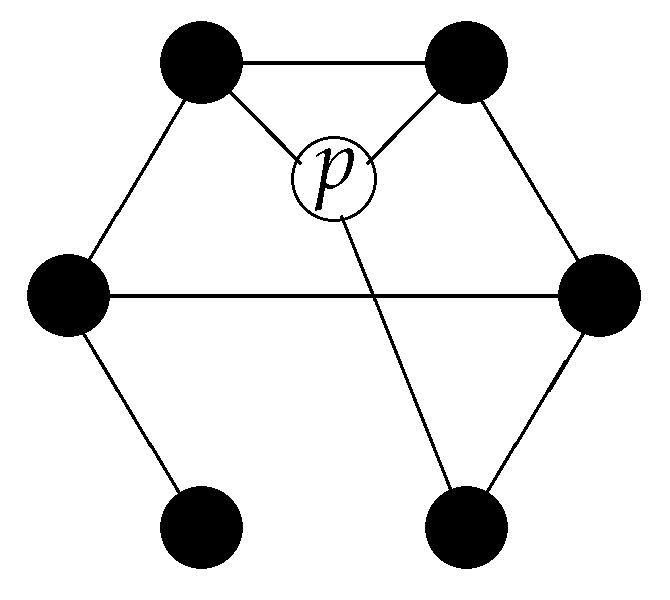
The location of an object *p* in a relational structure is defined by the spatial points to which it is related. Equivalently, one can specify this relation by the characteristic function of this set of spatial points. If the relations of the object to ‘space’ are directed, we can specify it by two characteristic functions.

In an absolute space, the location of an object is defined by x(t), i.e., by specifying the spatial point *x* *at* which an object is at this particular moment. In a relational space, the location of an object is defined by a field χ(x,t) (x∈V), indicating the relations of this object to the spatial points (see Figure 2). For an undirected mathematical relation, this field only assumes two values, 0 and 1, depending on whether or not the relation exists, while, for a directed mathematical relation, this field can be considered as having two binary components specifying the “in”- and “out”-relations.

**Figure 3:**
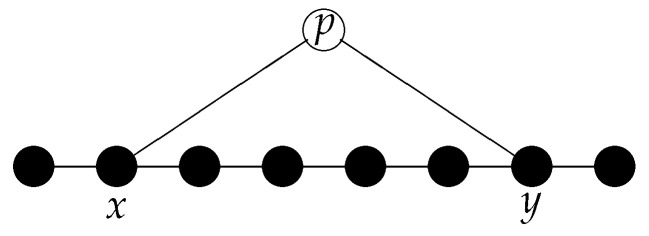
In a relational framework, a particle can be at two locations simultaneously. In the given example, the object *p* “is” at the points *x* and *y* simultaneously.

In a relational space, it is also possible that an entity can be “in two different spatial regions simultaneously” (for an example, see Figure 3).

More general, if an object *p* has relations to many spatial points that are distributed over a region with large distances, we may say that this object is non-local.

### 3.4. Complex Valued Relations

Up to now, I have considered “yes-or-no”- relations, defined by binary functions χ:V×V→{0,1}. For the corresponding graphs, a line between two points is either present or not. However, one can generalize this concept by attributing weights or distances or other quantities to lines. In this way, one arrives at the notion of a network. (For me, a network is a graph with additional structures; however, not all authors make this distinction.)

In quantum mechanics, the state of a particle at a particular moment *t* can be characterized by its wave function ψ(x,t), which is a complex-valued field over space. In the next section, I will distinguish between the relations among spatial points and the relations between some entity (“particle”) and spatial points. For the latter, the relations are defined by the wave function. However, here I only want to emphasize that, in general, complex-valued relations are also possible.

The following examples of networks, in which the relations between nodes can be generalized from a binary value (being there or being absent) to complex values, just serves as an illustration that networks with complex-valued relations exist. In most of the examples, a link between two nodes is used for an exchange of information (in the broadest sense) or activity between the nodes. This information or activity can be coded as a complex number for at least two reasons: either because there is a flow in both directions, from node *x* to node *y* and vice versa, or because the activity has an amplitude and a phase. From classical wave models, it is well known that these two possibilities are not completely independent.

As a first example, consider a network of computer servers (this example will be extended in Section 4.5). In this case, two servers are said to be “related”, if there exists a direct connection from one of the servers to the other. For a particular server *p* inside such a network, we can define a complex-valued, time-dependent relational structure by a function $\psi_{p}\left(q;t\right):V\to C$ (where *V* now denotes the set of computers), which characterizes the information exchanged between server *p* and another server *q*. As such, a function exists for all servers in the network, and we end up with $\psi \left(p,q;t\right)\equiv \psi_{p}\left(q;t\right):V\times V\to C$.

Another example of networks in which the relations between the constituents can be described by a complex-valued function are electric circuits with resistors, capacities and/or coils—the “relations” between nodes being the currents.

A different type of network, which I will sometimes use as an example, are neural networks (for an introduction to neural networks, see, e.g., [26]). One can define several types of complex-valued relations in such networks:

- In a neural network, the directed link between two nodes (which in this case are referred to as neurons) has a weight (the synaptic weight) which determines the transmission intensity of a signal. Negative weights indicate inhibitory influences. As the network is directed, the connection between two nodes is specified by two real-valued weights that can be combined into a complex number. These weights change over time as a result of learning.
- In so-called spiking neural networks, the signal consists of a firing rate (the number of spikes per unit of time) that is transmitted from one neuron to another. The time scales on which these firing rates change are much shorter than the time scales for changes in the synaptic weights, so that the synaptic weights can roughly be considered as constant. The connections (the synapses) between neurons are directed, but it often happens that connections exist in both directions. In addition, firing can occur in a synchronized way between clusters of neurons or asynchronous. Thus, the relative phases in spiking neurons can be important.
- On large scales (averaging over several hundreds of neurons), the activity in neural networks is sometimes described by a complex field (see, e.g., [27,28]). Together with David Bohm(!), the famous neuroscientist Karl Pribram developed a quantum field theoretic approach to consciousness [29], which was related to Bohm’s ideas of an implicate and explicate order [30].

## 4. One-Particle Quantum Mechanics

We know that quantum mechanics, based on Schrödinger’s equation, is only a non-relativistic limit of a theory that is considered to be more fundamental: quantum field theory. In Section 6, I will indicate how quantum field theory might be formulated in a relational setting. In this section, however, mainly for didactical purposes, I indicate how a relational re-interpretation of quantum mechanics might lead to an ontology of quantum theory.

### 4.1. The Generalized Relational Structure of “Location”

As we have seen in the previous section, in a relational framework, the position of an object is defined by the spatial points to which it is related. One of the consequences is that an object can be “at several spatial points simultaneously” (see Figure 3). Exactly this feature is one of the conundrums in the standard formulation of quantum mechanics. The wave function ψ(x) of a particle does not mark a particular point of space as the position of that particle, but it defines a whole region of space in which the particle, if measured, can be found. According to the general interpretation of quantum mechanics (not Bohmian mechanics), this uncertainty in the position of a particle is not due to a lack of knowledge but intrinsic. An object like an electron “presents” itself as a particle, when a proper measurement is performed.

There are many ways to combine a discrete model of space(-time) with quantum mechanics (a by far not complete selection of approaches can be found in [31,32]). In the following, I will describe just one possible model (more details can be found in [17,18,19]). In this model, the connection between wave mechanics and a relational model is made by generalizing the concept of a relation.

In principle, the relational structure among spatial points can be anything which in a large-scale limit gives rise to the topological and geometrical properties of our three-dimensional space. However, for simplicity, I still assume the relational structure among spatial points as represented by an undirected graph, i.e., for two spatial points *x* and *y*, a connection is either present or absent. Even this simple structure can in principle yield the desired large-scale limit.

In the micro-relational interpretation, the relations between an object (“particle”) and the set of spatial points will be generalized from a binary function to a complex-valued function ψ:V→C, and this complex function is the wave function of this object. Again, the micro-structure is not necessarily fixed: the requirement is that, in a large-scale limit, the relational structure between objects and spatial points yields the wave function; however, for simplicity, I assume that the micro-relations already have the complex values of the wave function. That such complex-valued relations can occur even in classical systems has been indicated in the last section.

I want to emphasize that, in the framework discussed here, the relational description of a single particle does not require a new mathematical formalism as compared to standard quantum theory (apart from the discretizations of space and time—and even these are not required). It is simply a different interpretation of the usual concept of a wave function (this will also hold for many-particle systems). In this re-interpretation, the absolute value of ψ(x), i.e., $p\left(x\right)={\left|\psi \left(x\right)\right|}^{2}$, should still give the probability(density) for finding a particle in a particular location when a measurement is performed. The changes with respect to the standard interpretation of quantum mechanics are minor: instead of speaking of a “probability amplitude” ψ(x), I refer to this function as a complex-valued relation. When this relation is probed by a measurement, it changes according to a “winner takes it all” manner (the “collapse” of the wave function). I will come back to these points in Section 4.4.

### 4.2. The Dynamics of Relations

For a relational structure between an object (particle) *p* and spatial points, one can define the dynamics as follows. First of all, as we are considering a discretized space, we also discretize time and formulate the dynamics in terms of an iterative mapping which defines $\psi_{p}\left(x,t+1\right)$, the $\psi_{p}$-function at time-step t+1, as a linear function of $\psi_{p}\left(x,t\right)$. (I use the notation $\psi_{p}\left(x\right)$ to indicate that this is the wave function of an entity *p*.) A natural candidate for such a dynamics is the following equation:

(2) $$\psi_{p}\left(x,t+1\right)=\psi_{p}\left(x,t\right)+\epsilon \left(\alpha \left(\sum_{y}A_{xy}\psi_{p}\left(y,t\right)\right)+\beta V\left(x\right)\psi_{p}\left(x,t\right)\right)\;.$$

The second term on the right-hand side (proportional to a constant ϵ) corresponds to the change of the generalized relation $\psi_{p}\left(x,t\right)$. There are two contributions: the first one describes the propagation of the relation from one spatial point to a neighbored point (expressed by the adjacency matrix $A_{xy}$), the second one describes an additional change of the relation due to a local potential. This second term may also depend on the valency or degree $d_{x}$ of point *x*, i.e., the number of points it is related to. It is not hard to see that, under very general conditions, such an equation becomes a Schrödinger-type equation in a continuum limit. Notice in this context that, for undirected graphs, the matrix D−A (where *D* is a diagonal matrix—the so-called degree matrix—with $D_{xx}=d_{x}$) is the graph Laplacian, i.e., the discretized analogue of the Laplace operator (see, e.g., [33]).

**Figure 4:**
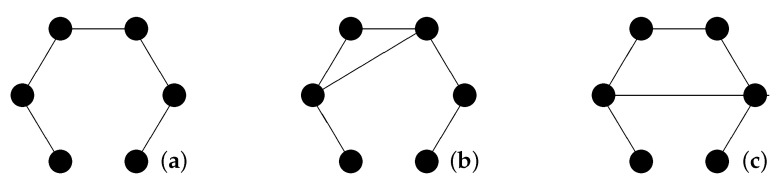
Propagation of relations: only via the intermediate step (**b**) can the additional relation in (**c**) be created from the relational space (**a**).

This specifies the dynamics of the relations of an object to the network of spatial points. However, how do relations change in general, e.g., how does the adjacency matrix $A_{xy}$ of spatial points change? (Changes in the purely spatial relations imply changes in the geometrical properties of space, i.e., they may become relevant if we include gravity.) At this stage, I introduce a locality requirement: If *x* and *y* are not related at time *t* (i.e., (x,y)∉E), they can only become related at time t+1 if there exists a point *z* such that (x,z)∈E and (y,z)∈E at time *t* (see Figure 4). This locality requirement is not mandatory, but it is quite satisfying from a philosophical point of view. As we will see later (Section 5.3), quantum correlations become local in this picture.

One may add further requirements, e.g., that an existing relation (x,y)∈E at time *t* can only be removed at time t+1, if there exists a point *z* such that either (x,z) or (y,z) have changed their status as a relation from t−1 to *t*. This “deletion” of relations may become relevant in the context of the measurement problem (see Section 4.4).

### 4.3. The Double-Slit Experiment and “Sum over Histories”

**Figure 5:**
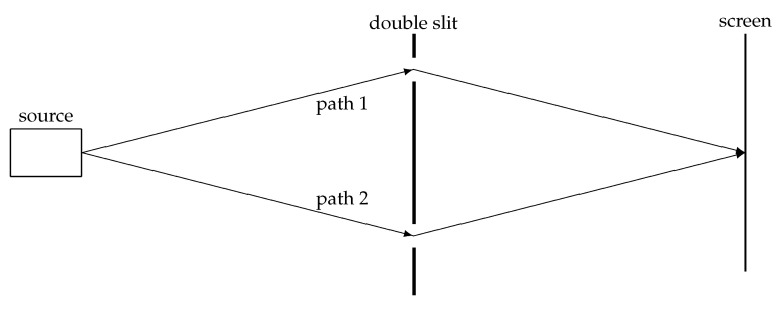
In the double-slit experiment, the total amplitude can be obtained by assuming that *aparticle* propagates along path 1 AND path 2. In the micro-relational interpretation, *the relations* of a particle propagate along path 1 and path 2.

For many scientists, the double-slit experiment is “the only mystery” of quantum mechanics (see, e.g., [34]). This may be arguable (in particular in view of the “mystic” effects related to entanglement), but the double-slit experiment has always been one of the paradigms of quantum theory. Therefore, it might be of interest to see how the double-slit phenomenon is explained in the relational setting (see Figure 5).

The interference pattern is easily explained by assuming a wave ψ(x) to propagate through the slits. The two parts of the wave behind the slits interfere and the intensity on the screen is obtained by the absolute square of the sum of these two parts. In a particle picture, Feynman’s “summation over paths” can be interpreted as: a particle propagates along path 1 with an amplitude $\psi_{1}$ associated with this path, and it also propagates along path 2 with an amplitude $\psi_{2}$ associated with this process. The absolute square of the sum of these two amplitudes yields the probability of finding the particle at a particular spot on the screen.

In the micro-relational interpretation, we can re-interpret the “summation over paths” in the following way: the entity *p* (the particle) has relations that propagate along path 1 and it also has relations that propagate along path 2 (compare Figure 3). The absolute square of the sum of these (complex-valued) relations yields the probability of finding the particle at a particular spot on the screen.

More generally, we can re-interpret Feynman’s “summation over paths”-representation for the propagator of a particle as “a relation propagates along path 1 AND a relation propagates along path 2 AND ...”. This seems to be much less weird than “the particle propagates along path 1 AND it propagates along path 2 AND it propagates along path 3 ...”.

### 4.4. Measurements

With respect to measurements, two fundamental concepts of quantum theory have to be explained: (1) probabilities are given by the absolute square of the scalar products of vectors that represent states, and (2) quantum states “collapse” into a new state (depending on the outcome of a measurement) as the result of a measurement. The first concept is known as Born’s rule, the second is sometimes called the “collapse” postulate. With respect to Born’s rule, we are mainly interested in probabilities for finding an entity at a spatial point (or in a spatial region), i.e., in probabilities proportional to $|\psi_{p}{\left(x\right)|}^{2}$.

Of course, we can simply postulate a mechanism which respects these two rules. Nobody will deny that it is easy to program a computer (essentially a classical system) to calculate probabilities from the absolute squares of complex functions and to update these complex functions according to the collapse postulate. However, I would like to include some examples of classical systems in which similar rules can be found.

In oscillating systems like the harmonic oscillator or simple waves, energies and intensities are given by the square of an amplitude. This also holds for alternating currents and voltages (the energy and electrical power being proportional to the product of these two). However, also in other processes, we encounter this relation: for instance, in diffusion processes or Brownian motion, the probability of finding a particle in a distance *d* from the origin of propagation is proportional to $d^{2}$. Thus, if a process is triggered by this particle (or by an intensity exceeding a given threshold) and if the ‘relations’ correspond to inverse distances, this process is triggered with a probability proportional to the square of these relations.

In addition, the collapse postulate is not completely unknown in classical physics: in some neural networks (e.g., in so-called Kohonen networks, see, e.g., [35]) the first neuron, which starts to fire as the result of an integrated input, sends inhibitory signals to all other neurons such that these will not fire. This mechanism—the first firing neuron inhibiting all other neurons—is sometimes called the “winner-takes-it-all” principle.

### 4.5. An “Every-Day” Example for Measurements and the Collapse

Instead of elaborating on possible realizations of the measurement process, I describe an every-day example which at first sight seems to have nothing to do with quantum theory. I hope that, in the end, the relationships will become obvious. This example relates to an example that has already been mentioned in Section 3.4.

You book a flight. What you get is an e-ticket. The essential information on that e-ticket is your name and the e-ticket number. Of course, it also tells you the flight number, the date and time of departure, the duration and additional information about your flight. What you need before you can enter the plane is a boarding-pass, which assigns to you your seat in the plane. The information that transforms your e-ticket into a boarding-pass is stored in some server at the airport or the airline.

**Figure 6:**
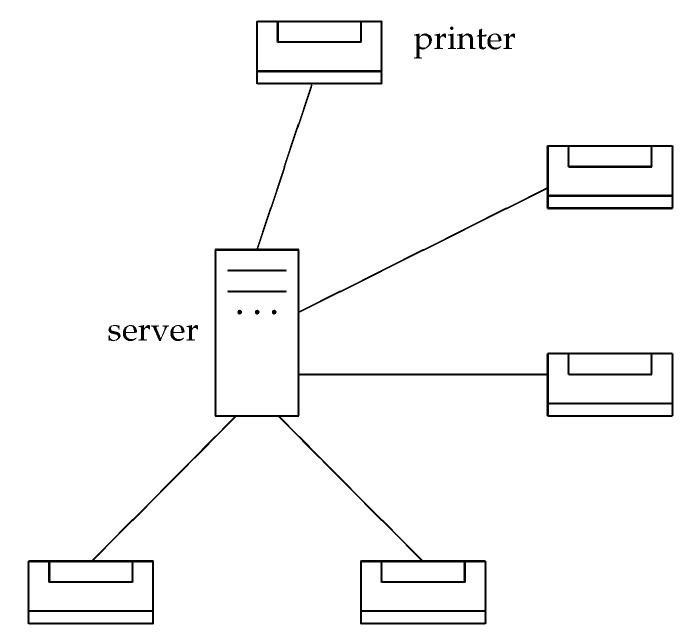
A server connected to a periphery of counters with printers is a model for a measurement in a relational system. A boarding pass exists only virtually as a program instruction in the server. Only when an e-ticket number is presented at a counter—this is the measurement—does the boarding pass become reality at the printer of this counter.

Before things get too complicated, I consider a simplified system (which comes close to the situation a few years ago). There is a single server that has the information about your e-ticket. Distributed over the airport are several counters with printers (Figure 6). When you go to one of the counters and present your e-ticket number, you will get your boarding-pass.

This boarding pass exists only once. You cannot go to a second counter and get a second boarding pass (you might get a second print-out, but it will be for the same seat number in the plane; in this sense, you can never get a second pass). In addition, when you go to a counter you never get “half a boarding-pass”, or part of a boarding pass, and, for the rest, you have to go to a different counter. Its an all or nothing situation, and the “all” can only happen once.

In order to make the situation more similar to quantum theory, let us assume a fictitious world in which you will get your boarding-pass at a particular counter only with a certain probability. If at one counter you do not get it, you have to try a different counter, and again you will get it only with a certain probability. The server decides probabilistically at which counter you will get the boarding pass, but it will set this probability to zero for a counter where you already unsuccessfully tried to get your boarding-pass (and it will renormalize the probabilities for the other counters). Eventually, you will get your boarding-pass at one of the counters.

The similarity to quantum theory should now be obvious: the boarding-pass is an entity that exists only “virtually” before it becomes reality as the result of a measurement. The measurement consists of the presentation of your E-ticket at one of the many counters. Before you make this “measurement”, the boarding-pass existed as a “potentia” (a virtual entity) at all counters simultaneously while, upon making the measurement, it becomes reality at only one of the counters. The counters represent certain locations where the boarding-pass can become reality. They correspond to the (discretized) spatial points.

Of course, you could come to the airport with your family and maybe many friends and present the e-ticket number at all possible counters simultaneously. Only at one of the counters will one of the members of your group get the boarding-pass.

## 5. Many-Particle Systems and Entanglement

### 5.1. General Remarks

Many-particle systems are a general problem for ontological theories. The many-particle wave function is defined in configuration space, i.e., in a 3N-dimensional space. Thus, in contrast to electric and magnetic fields or the metric field of space-time, which also “guide” particles, this field does not have an ontology in ordinary space. This feature is often used as an argument against Bohmian mechanics. The counter-argument is that, for a system of *N* interacting particles, the potential in Newtonian mechanics is also defined in configuration space, and the corresponding force acting on one particle may depend on the positions of all other particles. Factorization only occurs for external potentials or forces acting on single particles and being independent of the positions of the other particles.

Before I elaborate further on this subject, I take up the example of the last Section 4.5. A server at an airport can not only handle a single boarding-pass but thousands of e-tickets and boarding-passes simultaneously. If you have made a booking for two persons, you can instruct the server to hand over both boarding-passes at the same counter. Such correlations among the probabilities, at which counters the boarding passes “come into reality”, resemble entanglement correlations. As long as there is an information exchange within the server, entanglement correlations are no miracle. Even if there are several servers handling the boarding passes at an airport, there is no miracle if the information is shared by these servers, i.e., if there is an information exchange between them.

This example can be taken as a hint that entanglement correlations are the result of an immediate exchange between entities. These entities have to be directly related to each other. In other words, a relation between two entities can exhibit itself as an entanglement between these entities.

### 5.2. Relations for Two-Particle Systems

**Figure 7:**
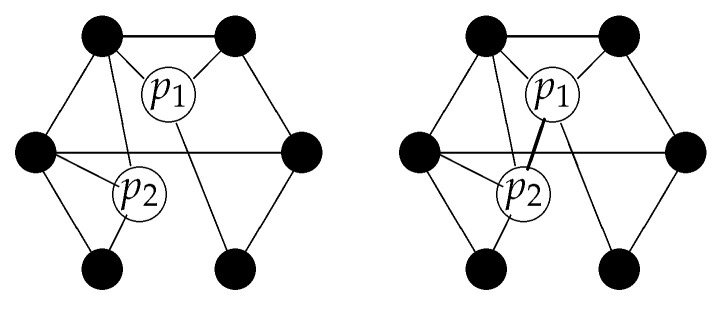
(**left**) two objects in a relational structure. Each object has its own set of relations to the spatial points. The relations factorize; (**right**) there can also be a direct relation between the two objects. This may lead to entanglement.

The simplest way to extend the one-particle picture to a two particle picture is to add two elements to the set of spatial points *V*, so that the set of elements now is $\{p_{1},p_{2}\}\cup V$ (see Figure 7).

The generalization of this construction to several particles is straightforward. For *n* particles, the relational space consists of the elements $V_{n}=\left\{p_{1},\ldots ,p_{n}\right\}\cup V$ and a generalized relation is a subset of $V_{n}\times V_{n}$. In Figure 7, the relations are undirected, but, depending on the nature of these relations, some of them may also be directed.

**Figure 8:**
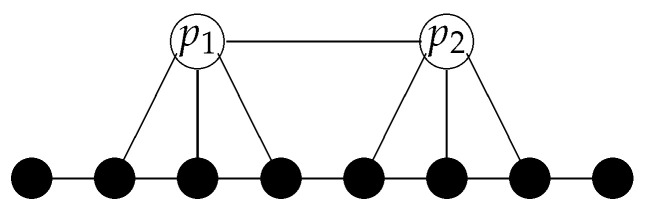
When there are several objects (in this case two), we have three different types of relations: (1) relations among spatial points, (2) relations between “particles” and spatial points, and (3) relations between the “particles”.

We now have encountered three types of relations (see Figure 8):

1. Relations between spatial entities: these are considered to be non-directed and give rise, on a large scale, to the geometry of space.
2. Relations between “particles” and spatial entities: these relations maybe directed and give rise, on a large scale, to the wave function.
3. Relations between “particles”: These relations are present if the particles are entangled. They allow for a direct information transfer between particles and characterize the form of entanglement.

When we compare this picture (e.g., Figure 7 or Figure 8) with our metaphor of servers and counters (or printers), the counters correspond to the spatial points *where*, in certain measurements, particles (boarding-passes) can be found. The algorithm that is stored in the server and which upon the presentation of the e-ticket sends the printing command to the periphery corresponds to the “virtual” entity before a measurement. The server (or the net of servers) just handles these virtual “many-particle algorithms”. Relations between particles, i.e., entanglement, can be compared with certain constraints between the different algorithms, and relations between particles and spatial points can be compared with connections between servers and printers (allowing for a selective output of the boarding pass at exactly one of the printers). In our metaphor, we do not take into account direct relations between printers. Such relations would define a spatial “neighborhood” and eventually give rise to a topology and a geometry on the set of printers.

There is an interesting point here: if entangled entities can exchange information, what mechanism restricts the degree of entanglement correlations to the Tsirelson bound [36] (see, e.g., [37,38,39])? There is no reason why correlations between systems that can exchange information are subject to a constraint that is much below the maximum possible correlation (Popescu–Rohrlich (PR) boxes [40] have maximum correlations), and it is easy to construct classical machines for which the correlations assume this PR-bound (see [41]). Of course, if the information exchanged is tailored according to the quantum formalism, this bound will be respected. However, it remains a general question why quantum correlations are subject to this bound.

### 5.3. Local or Non-Local, That Is the Question

One of the more speculative consequences of a relational space and, in particular, relational locations of objects in such a space, is the possibility of an ostensible superluminal propagation of influences (changes in relations) in the sense that actually this propagation of influences is subject to a locality principle (see Section 4.2), but, for an observer, it may look like an immediate, non-local influence or change of relations.

Of course, much depends on how we measure distances between the elements of relational sets. As already mentioned, apart from the simple mathematical concept of distance (number of links for the shortest path connecting two elements), one may use a propagator distance, which involves a summation over all paths connecting two elements. In [17,18], I have dealt with the consequences of such definitions.

Here, I would like to emphasize a slightly different point of view. Let us assume that distances in space are determined exclusively by the spatial relations and that these relations remain constant (e.g., consider a three-dimensional hypercubic lattice). Now, consider the situation of Figure 8: two entangled particles, each having relations to spatial points in regions that might be far away from each other. However, due to entanglement, these particles are directly related and can therefore “communicate” almost instantly. Two objects, which are entangled, are “nearest neighbors” and never far away from each other in the sense of relations. (There is a a similarity to the ideas behind the so-called ER = EPR conjecture of Maldacena and Susskind [42]: Two particles which are entangled (EPR, Einstein–Podolsky–Rosen entanglement, [43]) are connected by an Einstein–Rosen (ER) wormhole.)

There is a curious observation that supports this general idea: entanglement is always built-up locally; however, it can be destroyed non-locally. In order for two distant objects to be entangled, they either were directly (locally) involved in an interaction in the past (e.g., they were created in a decay process) or one of them interacted locally with a particle that already was entangled with the other (entanglement swapping). Both are local processes according to the definition given in Section 4.2. However, if two distant particles are entangled, this entanglement relation can be “broken” (they become separated) by a local interaction (e.g., a measurement) performed at only one of the particles. This asymmetry with respect to entanglement creation and entanglement destruction is nicely explained in the relational structure.

Violations of Bell’s inequalities are sometimes taken as a proof that any ontological model of quantum theory has to be non-local. Only seldom is it explicitly stated that this conclusion is based on a classical (non-quantum) picture of space-time, e.g., a Minkowski space-time as a background. The ER = EPR conjecture as well as the micro-relational interpretation circumvent this assumption.

## 6. Relational Space-Time—Relational Events

The previous sections assumed a relational space and a relational notion of “location” for an object in such a space. In this section, I will briefly sketch a relational structure of space-time.

When dealing with space-time, the relevant “objects” (the elements of space-time) are events. If space-time is considered as “absolute” (e.g., Minkowski space-time), the events are located at particular space-time points. In a relational picture, the locations of events (space-time points) are defined by their relations to other events.

**Figure 9:**
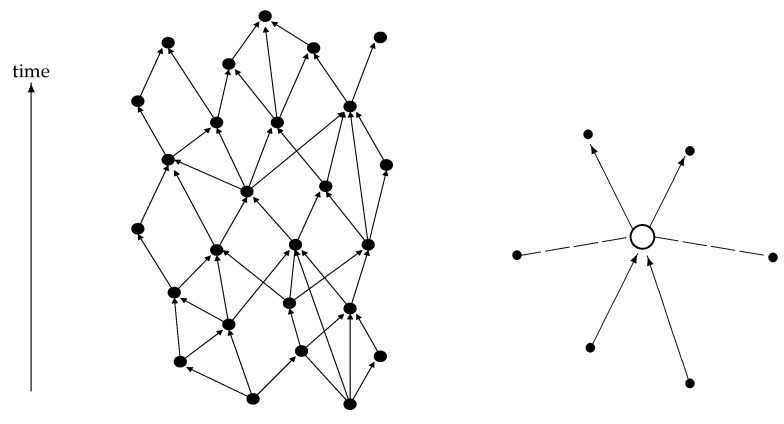
(**left**) the events making up the canvas of “space-time” are endowed with a causal structure; (**right**) a physical, object-related event can be related to the events of “space-time” in three different ways: It can be causally influenced by events in its past, it can influence events in its future and there may be “space-like” relations to events that are in the causal complement. The distinction between “space-like” events and time-like or light-like events depend on the real and imaginary parts of causal Green’s functions.

One starting point may be the model of causal sets (see, e.g., [44,45] and Figure 9, left). In this case, all relations are assumed to be time-like or light-like (depending on the details of the formalism). There are no space-like relations. The causal structure of space-time is built into the relational structure. I will assume such a relational structure for the space-time events that make up “empty space”, i.e., which in a large-scale limit approaches a Minkowski space or any other vacuum solution of Einstein’s equations.

With respect to the relations of an event like the emission of a photon by an electron, i.e., an event which involves entities like particles, I will choose a different structure. Having a quantum field theory in mind, I define space-like and time-like (including light-like) relations for object-related events (see Figure 9, right). The distinction between space-like and time-like relations will be that space-like relations are real-valued while time-like relations are complex-valued. The reason behind this definition is that, in quantum field theory, Green’s functions have real and imaginary parts for time-like separated points but only real parts for space-like separated points; and the relations that I associate to an event are defined by the Green’s functions.

**Figure 10:**
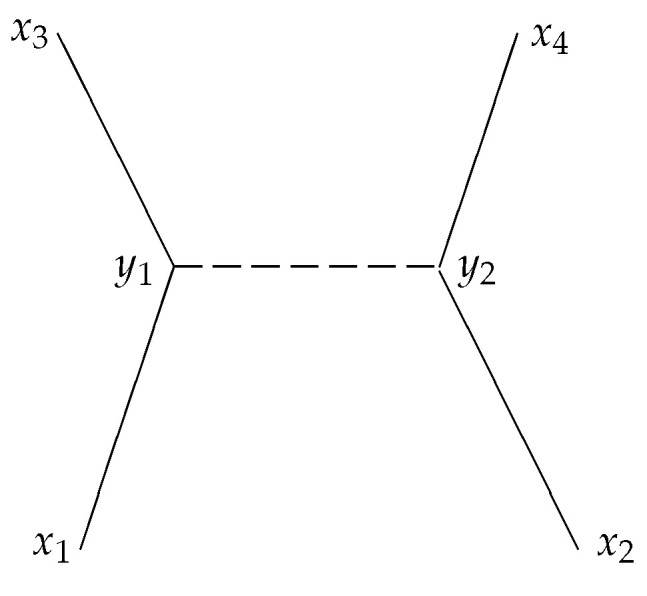
The lowest order approximation of a Coulomb scattering of two electrons by an exchange of a (virtual) photon. The points $x_{i}$ are kept fixed while one has to integrate over all possible positions of the intermediate events at $y_{1}$ and $y_{2}$.

Without going into details, I just consider the simple process of Coulomb scattering of two electrons in the lowest approximation (Figure 10). Two elementary events—the emission of a photon of one electron and the absorption of the photon of the other electron—constitute this process. Usually, the asymptotic states are characterized by their momenta, but, for simplicity, I consider the process as determined by four external events $x_{1},x_{2},x_{3},x_{4}$ that correspond to two initial states of the electrons and two final states of the electrons, respectively. Suppressing all indices referring to the spin of the electrons and the polarization of the photons as well as factors of π and other normalization factors etc., the amplitude for this process can formally be expressed as

(3) $$A\left(x_{1},x_{2},x_{3},x_{4}\right)\propto \int \;dy_{1}^{4}\;\int \;dy_{2}^{4}\;S\left(x_{1},y_{1}\right)S\left(x_{2},y_{2}\right)G\left(y_{1},y_{2}\right)S\left(y_{1},x_{3}\right)S\left(y_{2},x_{4}\right)\;.$$

Here, S(x,y) denotes the electron propagator (from space-time point *x* to space-time point *y*) and $G(y_{1},y_{2})$ the propagator of the exchanged (virtual) photon. In general, the contributions from these propagators are complex functions. Each propagator defines a generalized relation between the event (say $y_{1}$) and other events (in this case $y_{2}$, $x_{1}$ and $x_{3}$). The fact that we have to integrate over the “location” $y_{1}$ of this event indicates that this event does not happen at a particular point, but, in principle, everywhere in space-time. At least, this is the usual interpretation of this integration: we have to sum over all histories, i.e., all positions for these events. In the mircro-relational picture, this integration is interpreted as a “sum” over all relations which one event, say “emission of a photon”, has to all the other events of the space-time canvas. (Actually, as the exchange propagator for the photon between event $y_{1}$ and $y_{2}$ will not be on mass-shell, emission of a photon and absorption of a photon cannot be distinguished and should rather be interpreted as ‘interaction with a virtual photon’).

Thus, in the micro-relational interpretation, events do not have a particular location, but they have relations to all other events, space-time events and object-related events. The amplitude for a particular process in quantum field theory is just the remainder of the sum over all these relations. (For more details, see [18,19].)

## 7. Conclusions

I have argued that the concept of “locality” receives a completely different meaning when the positions or locations of entities (objects or events) are defined in a relational sense as compared to an absolute space or space-time. In particular, many counter-intuitive aspects of quantum theory appear less weird from this perspective. A relational space or space-time as well as a relational structure between particles might also be a way to circumvent the constraints given by Bell-type inequalities: the “elements of reality” and the requirement of locality are no-longer mutually exclusive.

I should add as a final remark that the ontological interpretation presented in this article is not necessarily opposed to Bohmian mechanics, at least not in the sense David Bohm interpreted his theory (see, e.g., [12]). The implicate order (or the structure underlying quantum theory and the theory of relativity) could be relational and the ideals outlined in this article may, in a large-scale continuum limit, lead to Bohmian mechanics.
